# Impacts of endometrioma on ovarian aging from basic science to clinical management

**DOI:** 10.3389/fendo.2022.1073261

**Published:** 2023-01-04

**Authors:** Zhouyurong Tan, Xue Gong, Yiran Li, Sze Wan Hung, Jin Huang, Chi Chiu Wang, Jacqueline Pui Wah Chung

**Affiliations:** ^1^ Department of Obstetrics and Gynaecology, The Chinese University of Hong Kong, Hong Kong, Hong Kong SAR, China; ^2^ Department of Obstetrics and Gynaecology, The Second Affiliated Hospital, The Chinese University of Hong Kong, Shenzhen, China; ^3^ Reproduction and Development, Li Ka Shing Institute of Health Sciences, The Chinese University of Hong Kong, Hong Kong, Hong Kong SAR, China; ^4^ School of Biomedical Sciences, The Chinese University of Hong Kong, Hong Kong, Hong Kong SAR, China; ^5^ Chinese University of Hong Kong-Sichuan University Joint Laboratory in Reproductive Medicine, The Chinese University of Hong Kong, Hong Kong, Hong Kong SAR, China

**Keywords:** endometriosis, ovarian preservation, premature ovarian insufficiency, treatment, target therapy

## Abstract

Endometriosis is a common reproductive disorder characterized by the presence of endometrial implants outside of the uterus. It affects ~1 in 10 women of reproductive age. Endometriosis in the ovary, also known as endometrioma (OMA), is the most frequent implantation site and the leading cause of reproductive failure in affected women. Ovarian aging is one of the characteristic features of OMA, however its underlying mechanism yet to be determined. Accumulated evidence has shown that pelvic and local microenvironments in women with OMA are manifested, causing detrimental effects on ovarian development and functions. Whilst clinical associations of OMA with poor ovarian reserve, premature ovarian insufficiency, and early menopause have been reported. Moreover, surgical ablation, fenestration, and cystectomy of OMA can further damage the normal ovarian reservoir, and trigger hyperactivation of primordial follicles, subsequently resulting in the undesired deterioration of ovarian functions. Nevertheless, there is no effective treatment to delay or restore ovarian aging. This review comprehensively summarised the pathogenesis and study hypothesis of ovarian aging caused by OMA in order to propose potential therapeutic targets and interventions for future studies.

## 1 Introduction

Endometriosis is a chronic inflammatory disease characterized by the presence of ectopic implants, including endometrium and granules, outside of the uterus. Its prevalence reaches 15% of reproductive-aged women and caused disturbances in their life quality due to severe pain and infertility (1). Endometrioma (OMA), the most common subtype of endometriosis, affects up to 44% of women with endometriosis worldwide (2). A strong correlation between OMA and infertility has been indicated in lots of prior studies to support the hypothesis that OMA *per se* and its treatments may imply quantitative and qualitative disturbance of ovarian reserve (3). OMA has been related to a lower ovarian reserve among infertile women, which is associated with ovarian aging and early menopause (4–7). Although the association is nonlinear and the underlying mechanism is obscured, molecular studies recently emerged to point out the potential mechanisms including iron accumulation, fibrosis, oxidative stress, DNA damage, genetics, and folliculogenesis interruption create a detrimental environment to impair follicle development and ovarian function, may subsequently lead to ovarian aging and early menopause (8–10).

The fertility capacity has long been known to diminish along with chronological age increase. In addition to natural aging, premature ovarian failure (POF)/primary ovarian insufficiency (POI) is defined as primary hypogonadism in women before the age of 40, characterized by interrupted folliculogenesis, reduced follicles, and interfered hormone production. POF/POI can also lead to premature ovarian aging manifested as early menopause and infertility (11–14). To date, many clinical studies demonstrated POF/POI could be induced by OMA and its treatments, crossing the bridge between OMA and ovarian aging. It is reported that patients with OMA who underwent surgical interventions have an increased risk of POF/POI [(15)]. It has been validated in animal models (16). Besides, it is addressed that hyperactivation of dormant primordial follicles, the onset of POF/POI, is induced by iron accumulation, fibrosis, and oxidative stress from OMA lesions (16). Despite the cause-effect relationship being under exploration, the investigations of the association and underlying mechanisms of OMA and ovarian aging are crucial for the development of potential therapies.

In this review, we thoroughly evaluated the clinical relationship between OMA and ovarian aging, summarized their potential mechanisms based on *in vitro*, *in vivo*, and clinical studies, as well as point out therapeutic targets, which may benefit the fecundity of infertile women with ovarian aging induced by OMA.

## 2 Clinical relevance of oma and ovarian reserve

Ovarian reserve is defined as the quality and quantity of the ovarian dormant primordial follicles. It determines the ovarian potential to provide functional eggs that are competent to fertilize (17). Only a limited number of primordial follicles are recruited to develop into growing follicles which either be selected for ovulation or go through atresia. Since most growing follicles are destined toward apoptosis and degeneration, only the left primordial follicles, remaining dormant in the cortex, reflect the ovarian reserve (18, 19). Ovarian reserve determines fecundity and fertility treatment success, its decline dictates the onset of ovarian aging (18, 20–22). Therefore, its assessment is pivotal for monitoring women’s ovarian function during ovarian aging and reproductive treatment. Due to the small size and lack of hormone secretion of primordial follicles, a clinical tool to directly assess primordial follicles does not exist (23). Currently available assessment tools for ovarian reserve, i.e., serum follicle-stimulating hormone (FSH), serum *anti-müllerian hormone* (AMH), and antral follicle count (AFC), only assess a small fraction of all follicles. Both FSH and AMH are predominantly produced by the developing follicles rather than dormant primordial follicles (24). Therefore the clinical value of serum FSH is limited for predicting ovarian reserve from a meta-analysis (25). The inaccuracy of serum AMH in resembling the number of primordial follicles was also confirmed when compared with ovarian cortical biopsies, which is a gold standard for ovarian reserve assessment (26). In addition, ultrasound detects AFC by identifying follicles with fluid-filled antrum, while it is limited to identify follicles in earlier stages and has difficulties in distinguishing healthy antral follicles from the ones undergoing atresia (27). The biopsy is a traumatic procedure that may decrease ovarian reserve and lead to other complications if not done properly (28). In summary, there is a lack of proper methods for assessing ovarian reserve accurately and safely. In this review, we analyze the effect of OMA on ovarian reserve according to the results from existing tools, but we also provoke for more suitable tools to be explored for an accurate assessment of ovarian reserve with less detrimental effects.

A meta-analysis presented that the ovaries with OMA had a lower AFC before and after surgical removal of lesions compared to the contralateral healthy ovaries, indicating both OMA itself and its surgical intervention can affect the number of growing follicles (29). Besides, a reduction in the serum level of AMH has been reported in women with OMA before surgical treatment (30). As a previous study has shown, there is a significantly lower serum level of AMH in women with bilateral OMA than the ones with unilateral or without OMA. Moreover, the extent of reduction is positively associated with the size of endometriotic lesions, indicating the OMA itself appears to be related to impaired ovarian reserve, and the effect depends on the size and bilaterality (31). Declined ovarian reserve after surgical removal of endometriotic lesions in patients with OMA has been widely reported in observational studies. A prospective cohort study evaluated the consequence of laparoscopic cystectomy of OMA on ovarian reserve and found a reduction in serum level of AMH postpone surgery (32). A decrease in ovarian reserve assessed by basal FSH and ovarian response during assisted reproductive technology (ART) treatment was observed in patients after surgical removal of bilateral OMA, and there is no correlation between the decline extend and the patient’s age (33). With the undisputable destroy effect of surgical interventions on the ovarian reserve of patients suffering from OMA and the advancement in modern surgical technology, some modifications were applied to the surgical process, including the choice of cystectomy or drainage, and the use of homeostatic agent during the surgical process. They were evidenced to improve fertility preservation to some extent but still cannot avoid the injurious impact of surgery (34–37). Recently, it is revealed that diminished ovarian reserve might be a consequence of premature primordial follicle activation, which is regulated by the phosphoinositide 3-kinase (PI3K)/*Protein kinase B* (Akt)/mammalian target of rapamycin (mTOR) and PI3K/*Phosphatase and tensin homolog* (PTEN)/Akt/Forkhead box protein O3 (FOXO3) signaling pathway (18, 38). The regulatory pathways are also proven to participate in the pathophysiology of endometriosis which thereafter leads to an increased rate of primordial follicle activation in ovaries with OMA (39–41). It can be verified among patients with unilateral OMA, whose density of primordial follicles in the ovarian cortex is lower in ovaries with OMA than the contralateral ones (7). Besides, the impact of surgical treatment on the activation of primordial follicles through the PI3K/PTEN/Akt/FOXO3 and mTOR signaling pathways has been addressed in both clinical and animal studies (42, 43).

There is limited evidence to show a direct relationship between ovarian aging and OMA, however, the hyperactivation of the primordial follicle in ovaries with OMA leads to ovarian reserve exhaustion, which therefore accelerates ovarian aging has been addressed in several studies (44). Since ovarian reserve in females decreased with chronological age in the natural aging process (45). It is reasonable to foresee that the prematurely primordial activation leading to loss of ovarian reserve may result in POF and subsequent ovarian aging. A research article demonstrated the PI3K/Akt/FOXO3 signaling pathway, which plays a main role in the primordial follicles hyperactivation in ovaries with OMA, is also important in females suffering from POF and the suppression of related pathways could improve the pregnancy rate in patients (46). POF represents the final stage of continuous loss of ovarian function and the absence of menstruation is one of the diagnoses of POF. Menopause represents the end of ovarian aging. However, the transitional process from a normal to absolute regression of ovarian function during the ovarian aging process caused by OMA has yet to be clarified (47–49). There is a commence of processes that took part in the pathogenesis of both OMA and ovarian aging, for example, oxidative stress, cytokines, DNA damage and repair, etc. On account of the limited studies to directly elucidate the association and underlying mechanisms between OMA and ovarian aging, extensive and longitudinal human studies are eagerly needed.

## 3 The mechanisms of OMA *per se.* leading to ovarian aging

### 3.1 Hyperactivation of primordial follicles and diminished ovarian reserve

The pool of dormant primordial follicles located in the cortical region of ovaries reflects ovarian function, which includes secreting ovarian steroids for homeostasis and producing qualified oocytes for fertilization (50). Folliculogenesis is a well-organized process that starts from primordial follicle activation to ovulation (51). Its disturbance during both physiological processes i.e., aging, or pathological diseases may result in a diminished ovarian reserve and impaired quality of oocytes (38, 51). Genome-wide microarray analysis of mouse ovary reported that adverse influence on folliculogenesis may contribute to the aging-dependent diminished ovarian function (52). Ovaries surrounding OMA were found with interrupted folliculogenesis, manifesting a decreased density of primordial follicles while a higher distribution of growing follicles compared with the contralateral healthy ovaries, which indicates a potential activation of primordial follicles in ovaries with OMA (53, 54). The initial activation of the primordial follicle is mainly under the regulation of the PI3K/Akt/mTOR, PI3K/PTEN/Akt/FOXO3 signaling pathways (18, 38). In addition, the Hippo/Yes-association protein (YAP) pathway is pertained to the process of primordial follicles by promoting oocyte development and granulosa cell proliferation (38). The involvement of PI3K/Akt/mTOR, PI3K/PTEN/Akt/FOXO3, and Hippo/YAP pathways were reported in both the pathophysiology of endometriosis and the physiological ovarian aging, uncovering its role in bridging OMA and ovarian aging (39–41, 55–57).

### 3.2 Fibrosis

In lots of tissues, especially the lung and liver, fibrosis is recognized in related diseases and induces organ failure. Characterized by excessive extracellular matrix (ECM) deposition and connective tissue elongation, fibroblasts developed and expand in response to constant tissue injury and inflammation, as well as physiological processes such as aging (58–60). With increased proinflammatory chemokines, immune cells, mostly M1 macrophages, are recruited to the damage site and trigger anti-inflammation which induces their differentiation to M2 macrophages. The M2 macrophages subsequently stimulate adjacent fibroblasts to produce collagen for scar formation and wound healing (61).

Increased collagen deposition has been demonstrated in the ovaries of women post-menopause as well as in the animal model of reproductive aging (62–67). A recent study documented that ovarian fibrosis originates from cellular stress-induced mitochondrial damage, which then leads to declined bioenergetics, oxidative stress, inflammatory mediators, and collagen accumulation, with reproductive aging. The fibrosis within the ovarian stroma results in anovulation, thereby causing fertility loss. The removal of fibrotic collagen from ovaries was demonstrated to prolong the female reproductive lifespan in mice (68).

The presence of dense fibrosis in the ovarian cyst’s pseudocapsule is widely known, which is also an important characteristic of ovarian OMA (69). One study revealed a higher fibrotic content in the ovarian endometriotic lesions compared to other subtypes of endometriosis (70). The expression of α-smooth muscle isoform of actin (α-SMA), which is pivotal for microfibroblast activation, was detected in ovarian cysts through immunostaining, suggesting myofibroblast proliferation (71, 72). Additionally, fibrosis was significantly extensive in ovaries with OMA compared to the contralateral healthy ovaries (53). Therefore, it is hypothesized that ovarian OMA affects the microenvironment and leads to fibrosis of the surrounding ovarian tissue. The presence of fibrosis in the ovarian cortex adjacent to OMA might furtherly result in a declined follicular density and decreased ovarian reserve, eventually acting as a causative factor for ovarian aging (70). The transforming growth factor-β (TGF-β)/Smad signaling pathway was found essential to the epithelial-to-mesenchymal transition of endometriotic cells derived from OMA, which is involved in the pathophysiology of OMA. Briefly, fibrosis formation can be accelerated by fibroblast-to-myofibroblast transdifferentiation and subsequent surge in collagen production and cell contractility. This process was proved to be reversed by TGF-β blockade (73).

### 3.3 Stiff and stretch

Tissue stiffening is one of the hallmarks of fibrosis. Increased stiffness accelerates the microfibroblast to produce collagen and then further promotes matrix stiffness, leading to a fibrotic microenvironment of surrounding tissues over time (63, 74, 75). Increased stiffness could activate EMT through myriad transcription factors predominantly TGF-β1. TGF-β1 also underlies the pathophysiology of endometriosis (76–78). Continuous stiffening of surrounding ovarian tissues by OMA lesions has been assumed to reduce ovarian reserve (79).

Stretch from OMA lesions may activate Yes-associated protein (YAP) and transcriptional co-activator with PDZ- binding motif (TAZ), the effectors in downstream of the Hippo signaling pathway, in the adjacent ovarian cortex (80). A meta-analysis compared the serum level of AMH in patients with OMA or benign ovarian cysts. Although the persistent stretch also presents in benign cysts, only the ovaries with OMA presented a significantly reduced serum level of AMH (30). While the reduction of AMH expression is independent of the size of OMA before surgical incision (81). Therefore, instead of stretching alone, there might be other factors that incorporate the mechanical stimuli to facilitate follicle loss in ovaries with OMA. An *in vitro* study demonstrated stretch and stiffness share a similar mechanotransduction mechanism, and there is an interconnected effect of stretch and stiffness levels on cell phenotypes (82). It is conceivable that stretch and stiffness co-ordinately induce mechanotransduction to activate YAP/TAZ.

The hippo signaling pathway acts as a downstream effector of Akt signaling and inhibits FOXOs and TSC1/TSC2 to activate mTORC1 (83–85). However, sole activation of Akt is insufficient to activate YAP/TAZ, mechanical stimulus such as stretch is also needed (85). The hippo signaling pathway regulates the activation of primordial follicles has been reported in mice (83). During *in vitro* activation of human follicles, a reduced expression of TSC1 and LATS1, inhibitors of Hippo and PI3K/Akt/mTOR signaling pathways, was demonstrated (43). This indicated that stiffness and stretch activate the related signaling pathways of primordial follicle activation and subsequently defect ovarian function.

### 3.4 Oxidative stress and cytokines

OMA cyst is proven to contain numerous toxic contents which could also trigger hyperactivation of primordial follicles and diminish the follicle density, thereby accelerating ovarian aging in patients with OMA. According to the molecular milieu of endometriotic cysts, a higher level of free iron was reported in both cyst wall and cyst fluid for a long time (86). Unlike combined iron which plays an essential role in several physiological activities, free iron mediates the generation of reactive oxygen species (ROS) and produces oxygen-free radicals through the Fenton reaction (87). The surrounding ovarian cortex is affected by excessive oxidative stress and presented a significantly increased expression level of 8-OHdG, a DNA damage marker, compared with benign ovarian cysts (88). Oxidative stress triggers hyperactivation of primordial follicles through the PI3K/Akt/mTOR signaling pathway in which PTEN is inhibited and oncogenes like Akt are activated (89). Meanwhile, myofibroblasts proliferation and collagen production are stimulated by excessive oxygen stress, therefore leading to tissue stiffening of the ovarian cortex similarly to the phenotype of senescence (90). Since the guanine bases, composing unit of telomeres, are vulnerable to oxidative damage and the oxidized lesions are inefficient to be repaired, oxidative stress is regarded as the main cause of telomere shortening, leading to reproductive senescence (91).

Numerous chemokines, cytokines, and growth factors arising from the OMA regulate PI3K/Akt/mTOR pathways activation and therefore have a potentially detrimental effect on follicle growth in the adjacent ovaries. Among factors involved in the pathophysiology of OMA, some molecules like vascular endothelial growth factor (VEGF) and interleukin (IL)-8 also participate in the PI3K/Akt/mTOR pathways (92, 93). The expression levels of proinflammatory cytokines such as tumor necrosis factor-alpha (TGF-α), IL-1, IL-6, and IL-8 are significantly increased in endometriotic lesions and fluids from patients with OMA (86, 94–96). The pivotal role of IL-1 in regulating folliculogenesis and the positive effect of IL-1β on the activation of primordial follicles were verified in *in vitro* culture system of the bovine ovary (97). The IL-16 promotes primordial follicle activation and development during *in vitro* culturing of the rat ovarian tissue (98). Recently, in an animal model of ovarian aging, the cytokines including IL-6, IL-8, and TNF-α were proved to contribute to the depletion of ovarian reserve (99). It adds plausibility to the concept that chemicals derived from OMA could result in ovarian aging although there is no direct evidence from both human studies and animal models.

### 3.5 DNA damage and repair

Various DNA damage agents can be continuously exposed to human beings which then impact health situations and modulate disease states (100). However, intricate and complicated systems in cells, involving DNA repair, damage tolerance, cell death pathways, and cell cycle checkpoints, faithfully protect DNA from deleterious consequences (100). DNA repair pathways are intrigued by the robust DNA damage response (DDR) followed by DNA damage. Major DNA repair pathways such as homologous recombination (HR), mismatch repair (MMR), nucleotide excision repair (NER), non-homologous end joining (NHEJ), and base excision repair (BER) are active along the cell cycle, permitting the DNA damage in cells (100).

Preserving genomic sequence information is pivotal for life perturbation. While the well-toned system of DNA damage/repair has been disrupted or deregulated along natural aging and many diseases, consequently, leading to declining fertility as the earliest phenotype of human aging (101, 102). With the advent of next-generation sequencing (NGS), alteration of proteins charging DNA recombination and repair have been screened in patients with POF (103–114). Minichromosome maintenance (MCM) 8 and MCM9 belong to the Mini Chromosome Maintenance family of proteins involved in HR (103). The lack of MCM8 leads to sterility and degenerated ovarian function in animal models (104). Consistent with the observation, a missense variant in MCM8 was found in the patients with POF and primary amenorrhea (105). Since HR initiates DNA double-strand breaks and is important for meiosis, the fibroblasts that come from the patient with POF were more sensitive to chromosomal breaks and the recruitment and activity of MCM8 at the site of DNA breaks were revealed to be impaired (105). Likewise, the absent expression of MCM9 in mice impairs meiotic recombination and oocyte generation (104). Pathogenic variants on MCM9 were also found to be responsible for POF in several studies (109–111). In addition, BRCA genes are involved in ataxia-telangiectasia-mutated (ATM)-mediated DNA double-strand break repairing (115). Mutations of BRCA1 and BRCA2 genes were found to boost reproductive aging and premature infertility (116).

ROS, the pivotal mechanism of OMA-related infertility, was reported to cause DNA damage *via* attaching DNA bases and compromising the DNA backbone (100, 117). A decreased expression level of copious genes implicated in DNA double-strand break repair, including BRCA1, was found in patients with endometriosis, and its level is positively correlated with ovarian reserves (118). Mammalian oocytes respond to extensive DNA damage at the prophase stage of meiosis through the activity of the DDR and Spindle Assembly Checkpoint (SAC) pathways (119, 120). It is verified by *in vitro* maturation of mouse oocytes with follicular fluid (FF) from women with endometriosis, in which the FF from patients with endometriosis upraised ROS levels in oocytes and switch on the DDR and SAC pathways. The sensitivity of oocytes to DNA damage checkpoints is increased and prevents oocyte maturation in women with endometriosis (121). A modulated DNA damage response was also observed in the eutopic endometrium of endometriosis, indicating the disturbance of DDR and DNA repair genes and their implication for impaired infertility in patients with endometriosis (122).

These studies highlight the role of pathogenic variants of essential regulators during DNA damage/repair in maintaining fertility in patients with ovarian aging and OMA. The therapeutic targets of related genes may have the potential to alleviate DNA damage and restore fertility potential for these patients.

### 3.6 Dysregulation of ovarian angiogenesis

Angiogenesis is a highly programmed process of growing new blood vessels from existing vascular structures (123). Active angiogenesis can be found in both physiological and pathological conditions in the reproductive organs of adult females, such as ovaries (124). Highly regulated angiogenesis is crucial for reproduction to support folliculogenesis and endometrial development (125). Dysregulation in angiogenesis, which generates extensive blood vessels, may contribute to the onset and development of many diseases (126).

Dysregulated angiogenesis plays a critical role in the pathogenesis of endometriosis as it enables the engraftment of endometriotic implants and their subsequent progression (127). Elevated expression of proangiogenic factors, such as vascular endothelial growth factor (VEGF)-A and hypoxia-inducible factor (HIF) -1/2α were found positively correlated to OMA, aiding in the growth of endometriotic lesions (128). Bevacizumab, an inhibitor of VEGF, showed no detrimental effect on ovarian reserve while suppressing the progression of endometriotic implants in a rat model of endometriosis (129).

Nevertheless, robust angiogenesis in ovarian follicles and corpus luteum (CL) was recently uncovered with an imaging system, confirming that the generation of new blood vessels was pivotal to guaranteeing a sufficient supply of nutrients and hormones during follicle maturation and development (130). It was reported that the administration of axitinib, the blocker of angiogenesis, decreased ovarian follicle consumption, postponed ovarian aging, and extended female reproductive longevity by suppressing follicle recruiting and development (130). It implied a potential clinical approach to pause lesion progression and delay ovarian aging in patients with OMA.

### 3.7 Genetics

**Figure 1:**
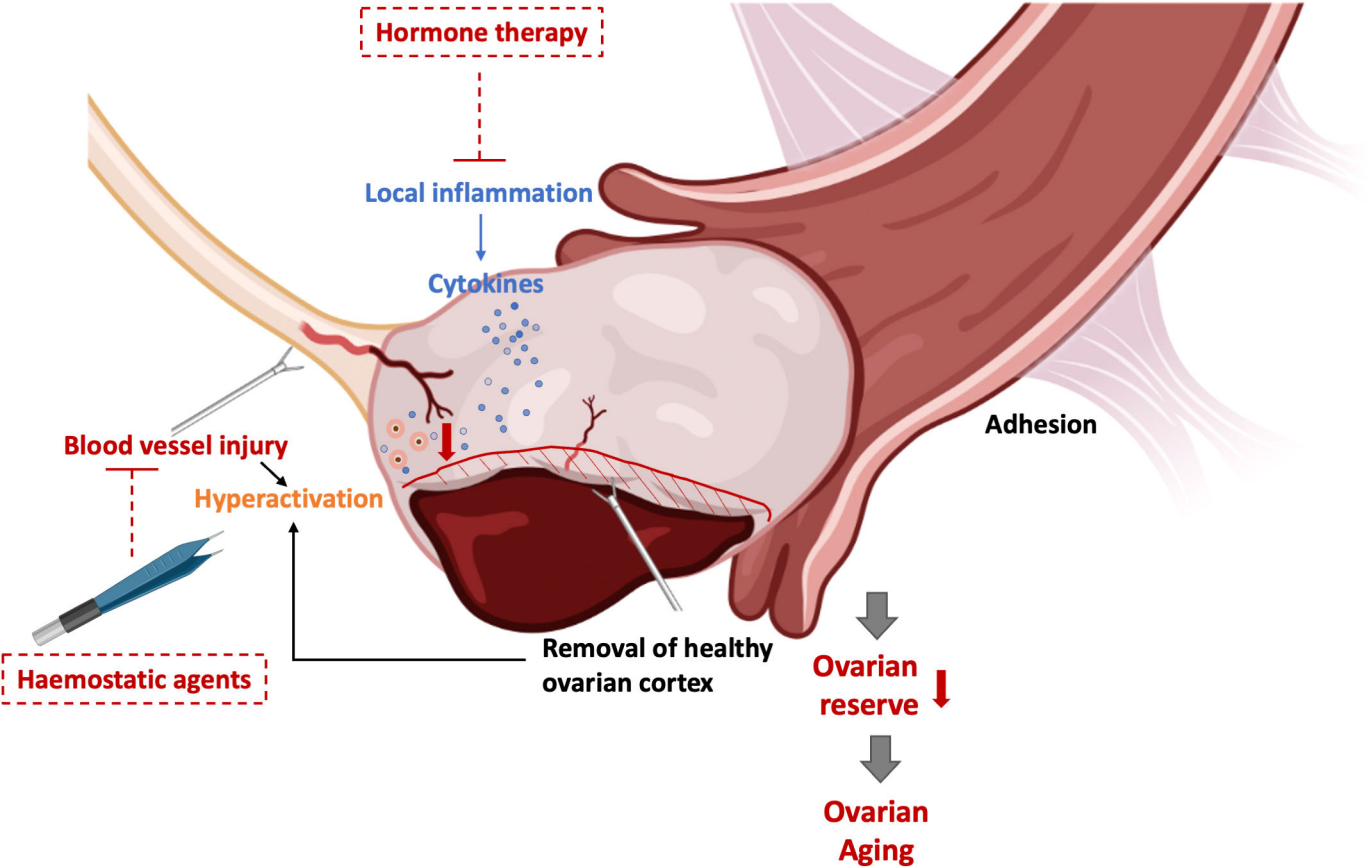
The potential mechanisms of ovarian aging in OMA per se and their potential target therapies. PI3K, phosphoinositide 3-kinase; PTEN, phosphatase and tensin homolog; Akt, Protein kinase B; FOXO3, Forkhead box protein O3; mTOR, mammalian target of rapamycin; YAP, Yes-associated protein; TGF, Transforming growth factor; FoxL2, Forkhead Box L2; GDF9, Growth Differentiation Factor 9; BMP15, Bone morphogenetic protein 15; VEGF, Vascular endothelial growth factor; IL, interleukin; POF, premature ovarian failure; POI, premature ovarian insufficiency.

Early menopause, the result of ovarian aging, can happen in women with a disrupted ovarian function which stop producing sexual hormones, especially estrogen. Age at menopause is estimated to be attributed 50% attributed to genetic factors (131). The presence of myriad genetic aberrations has been identified in POF genomics (132). Promising candidate genes like Forkhead Box L2 (FOXL2), growth differentiation factor 9 (GDF9), and bone morphogenetic protein 15 (BMP15) are also contribute to the pathophysiology of OMA (133–136). the overlapped genetic aberrations are hardly surprising since both OMA and POI potentially affect the formation of the ovarian reserve from the primordial follicle pool, disrupt oogenesis and meiosis, and lead to follicle dysfunction by interrupting folliculogenesis (11, 132). The insights revealed the essence of the genetic analysis point to potential new drug targets for improving fertility in women with OMA-related-ovarian aging. **Figure 1** presented the potential mechanisms of OMA itself which leads to ovarian aging.

## 4 The mechanisms of OMA treatment leading to ovarian aging

### 4.1 Tissue damages

Because of the ineffectiveness of medical therapies, there is a general consensus that OMA requires surgical treatment (137). There are various surgical methods including ablation, fenestration, cystectomy, etc. Thereinto, cystectomy by the stripping method is the most common method applied to patients with OMA, because of the lower recurrence rate and more favorable reproductive outcomes compared with others (138). However, OMA cysts are difficult to be removed without damaging surrounding follicular tissue (139). Cystectomy may also cause adhesion and injury to the surrounding blood vessel, which further impeded the development of growing follicles since they are extensively surrounded by blood vessels (140, 141). Moreover, the combination of bipolar diathermy will speed up the damage to the follicles since it literately burns out the follicles using thermal energy. In a word, it is hypothesized that the damage to surrounding tissue and blood vessels may result in impaired ovarian function and thereby speeds up ovarian aging (142).

The hypothesis is supported by numerous clinical studies. It was observed that after ovarian OMA excision, women’s responsiveness to hyperstimulation was reduced and the menopausal transition occurred earlier (15, 143). A retrospective crossover study examined the ovulation rate in 28 infertile patients with unilateral OMA to evaluate the result of ovarian cystectomy. It showed that the ovulation rate significantly declined in the affected ovary after laparoscopic cystectomy as compared with before surgery (144). A cohort study presented that compared with the control group, women with OMA had significantly lower AMH concentrations at baseline and exhibited a further reduction at 6 months postoperatively (145). A prospective randomized study evaluated women who underwent ovarian surgery to remove OMA underwent substantially longer stimulations and required substantially higher dosages of recombinant FSH compared with those who proceeded directly with IVF-ICSI. Additionally, these patients with OMA surgery had a substantially lower oocytes retrieval rate. However, for the fertilization and pregnancy rates, there was no observed difference (146). Surgery for OMA greater than 5 cm in diameter and bilateral OMA resulted in more extensive damage to ovarian reserve (33, 147). Overall the surgical incision of OMA potentially implies a detrimental effect on the surrounding ovarian tissue which subsequently boosts ovarian aging.

### 4.2 Hyperactivation of primordial follicles

The impact of surgical injury on primordial follicle activation has been determined in several studies. An *in vitro* study demonstrated that surgical injury to the surrounding ovary could activate dormant primordial follicles near the surgical incision through the mTOR signaling pathway (42). mTOR plays an important role in ovarian aging. It allows different types of cells to escape from the normal biochemical system and regulates the balance between apoptosis and survival (148). Furthermore, surgery could induce local inflammation. The triggered cytokines could affect primordial follicles and/or ovarian reserve in ovaries with resected OMA. for instance, IL-1α may play a pivotal role in the age-related exhaustion of the ovarian reserve in mice by promoting apoptotic pathways and enhancing the expression of pro-inflammatory cytokines IL-1β, IL-6, and TNF-α (149). In addition, a mouse study presented that lipopolysaccharide (LPS) accelerated primordial follicle activation through the PI3K/PTEN/Akt/FOXO3 signaling pathway (150). The activation of this pathway may lead to a compromised DNA damage response, then impacting the growth of primordial follicles and ovarian aging (55). An *in vitro* experiment illustrated that human ovarian fragmentation culturing resulted in immediate translocation of the Hippo/YAP into the nucleus of granulosa cells (43). In specific regards to the development of ovary tissue, and ovarian follicles, actin polymerization-enhancing drugs promote ovarian follicle growth mediated by YAP (151).

**Figure 2:**
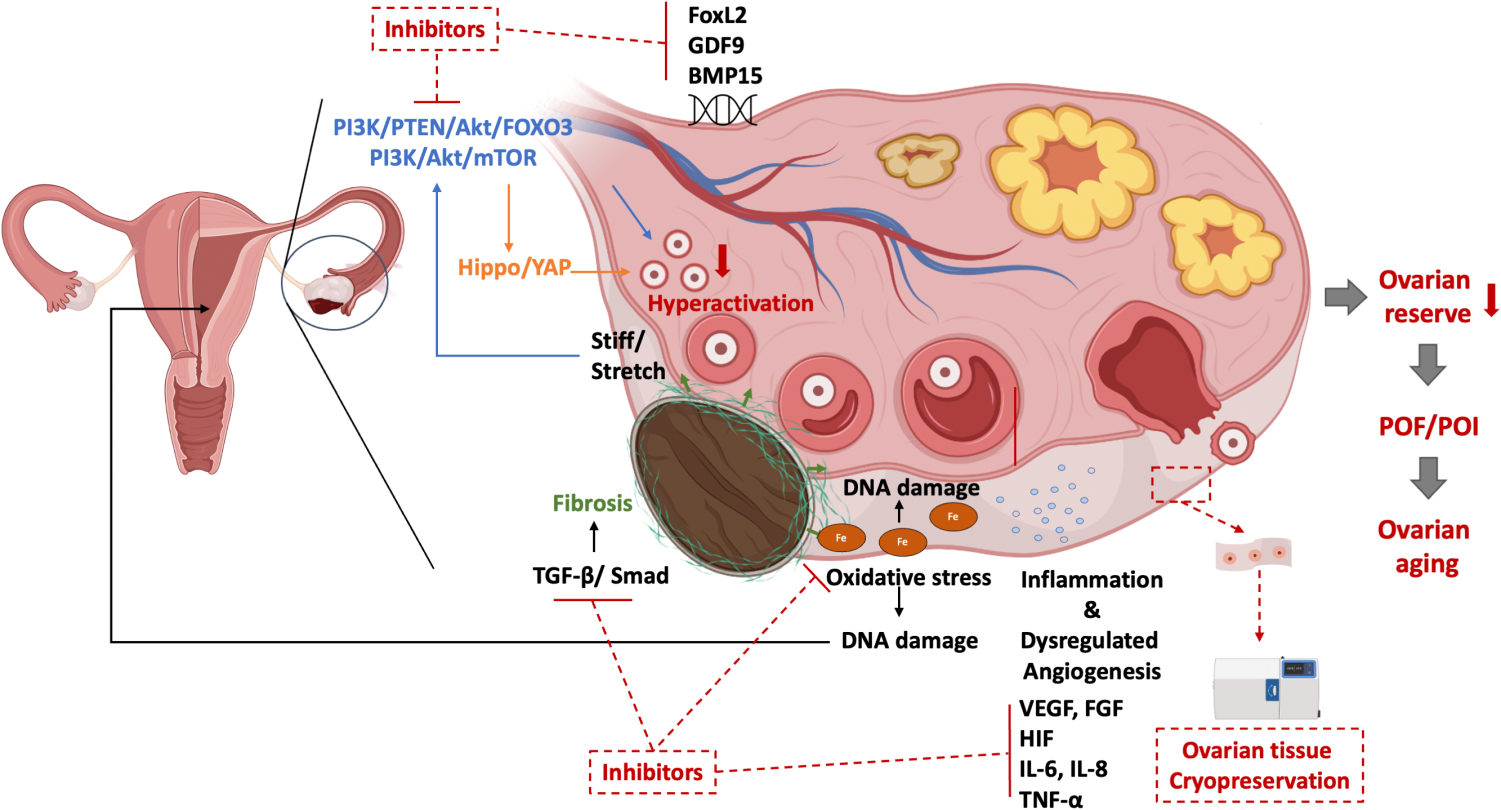
The possible pathogenesis of ovarian senescence due to OMA interventions and their treatments.

Taking all information together, when deciding whether a patient needs to go through surgery to remove an OMA, every clinician should not only consider symptom relief and recurrence of disease but also ovarian responsiveness, chances of conception during IVF cycles, ovarian reserve, and the possible tendency of ovarian aging. The possible pathogenesis of OMA interventions leading to ovarian senescence is illustrated in **Figure 2**.

## 5 New treatment and target therapy

### 5.1 Therapeutic targets

Therapeutic targets aim at reversing ovarian aging and thereby restoring fertility in the aspect of ovarian function are essential for the investigation and development of novel drugs. Based on the mechanisms of OMA leading to ovarian aging described in parts 3&4, 15 drugs targeting the related pathogenesis and signaling pathways were screened in the *Therapeutic Target Database* (**Table 1**). Some of them are already put on the market for other conditions, such as myeloma, pulmonary fibrosis, and diabetes, but still with potential applications in the area of OMA-induced ovarian aging from related studies. For instance, both *in-vitro* and *in-vivo* studies demonstrated that Sirolimus, which was approved for myeloma, induced regression of endometriotic lesions through inhibiting angiogenesis and proliferation (152–154). Anti-fibrotic agent Pirfenifone was proven to reduce postoperative adhesions for women with endometriosis (155). Siltuximab, an antiviral agent targeting IL-6, is one of the drug candidates for endometriosis-related infertility from the computational drug discovery (156). In addition, Menotropins stimulation may attenuate infertility caused by endometriosis and benefit the IVF-ET outcome (157). And Bevacizumab, an angiogenesis inhibitor, dramatically reduced the size of endometriotic lesions with no impairment to ovarian reserve (129). In account of the utilization in reproductive medicine, the pregnancy risk of the potential drugs is necessary to be considered. According to the guidelines proposed by the United States Food and Drug Administration, five-letter risk categories (A, B, C, D, and X) indicate the potential of a drug to induce birth defects if used during pregnancy (159). It is noted that Menotropins are classified as category X, and thus need to be applied under cautions and contraindicated if conception has occurred. Furthermore, some preclinical therapeutic agents are observed to reverse ovarian function in *in-vitro* and *in-vivo* models of OMA and aging. Quercetin, an antioxidant, delayed oocyte aging and improved the developmental potential of aged oocytes during *in vitro* culturing system (160). Application of ammonium trichloro (dioxoethylene-o,o’) tellurate (AS101), a modulator of the PI3K-PTEN-Akt pathway, was proved to preserve ovarian reserve in mice ovaries with OMA by inhibiting the hyperactivation of primordial follicles (41). It was first reported that ovarian fibrosis in reproductive-aged mice could be reversed with antifibrosis drugs (pirfenidone and BGP-15) and thereby improved female fertility (68). The insights revealed in these therapeutic agents point to a prospective application in treating women with OMA accompanied by ovarian aging. More preclinical and clinical trials should be launched for their further development.

**Table 1:** Potential drugs targeting the signaling pathways and molecules of OMA related to ovarian aging.

Signaling pathway/molecules	Drug	Drug Type	Indication	Therapeutic Class	Clinical Phase	Any study in endometriosis	Pregnancy Category
PI3K/AKT/mTOR pathway (PAm pathway)	Sirolimus	Small molecular drug	Multiple myeloma; Organ transplant rejection; Dutch elm disease	Immunosuppressive Agents	Approved	Rapamycin induces regression of endometriotic lesions (152–154)	C
Transforming growth factor beta (TGFB)	NIS793**^1^**	/	Pancreatic ductal carcinoma; Solid tumour/cancer	Anticancer Agents	Phase 2	/	/
Transforming growth factor beta 1(TGFB1)	Pirfenidone	Small molecular drug	Idiopathic pulmonary fibrosis	Antifibrosis Agents	Approved	Pirfenidone moderately reduced postoperative adhesions (155).	B3
Electron transport complex III (Complex III)	Tafenoquine	Small molecular drug	Malaria; Plasmodium vivax malaria	Anti-Parasites Agents	Approved	/	C
Reactive oxygen species (ROS)	Tafenoquine	Small molecular drug	Malaria; Plasmodium vivax malaria	Anti-Parasites Agents	Approved	/	C
Signaling pathway/molecules	Drug	Drug Type	Indication	Therapeutic Class	Clinical Phase	Any study in endometriosis	Pregnancy Category
Interleukin-1 alpha (IL1A)	MABp1**^1^**	/	Colorectal cancer; Acne vulgaris; Atopic dermatitis; Hidradenitis suppurativa; Peripheral vascular disease; Plaque psoriasis; Pyoderma gangrenosum; Type-2 diabetes	Anticancer Agents	Phase 2; Phase 3	/	/
HUMAN interleukin 6 (IL6)	Siltuximab**^1^**	Monoclonal antibody	Anemia, Multiple myeloma, Idiopathic multicentric Castlemans disease; Coronavirus Disease 2019	Antiviral Agents	Approved; Phase 3	Siltuximab is one of the potential drug treatments for endometriosis-induced infertility (156)	C
HUMAN interleukin 8 (IL8)	BMS-986253**^1^**	Monoclonal antibody	Coronavirus Disease 2019 (COVID-19)	Antiviral Agents	Phase 2	/	/
Tumor necrosis factor (TNF)	Infliximab**^1^**	Antibody	Plaque psoriasis, Asthma, Rheumatoid arthritis	Immunosuppressive Agents	Approved	Infliximab appears not to affect pain associated with deep endometriosis (158)	B
Follicle-stimulating hormone receptor (FSHR)	Menotropins	/	Female infertility	Fertility Agents	Approved	Menotropins stimulation may overcome some causes of infertility in patients with endometriosis (159).	X
Signaling pathway/molecules	Drug	Drug Type	Indication	Therapeutic Class	Clinical Phase	Any study in endometriosis	Pregnancy Category
HUMAN vascular endothelial growth factor (VEGF)	Bevacizumab	Monoclonal antibody	Metastatic colorectal cancer	Antiviral Agents	Approved	Reduce the size of endometriotic lesions, no detrimental effect on ovarian reserve (129)	C
HUMAN vascular endothelial growth factor receptor (VEGFR)	Axitinib	Small molecular drug	Renal cell carcinoma	Anticancer Agents	Approved	Under clinical trial (NCT03481842)	D
Hypoxia-inducible factor 1 alpha (HIF-1A)	HIF-1alpha^1^	Small molecular drug	Lymphoma	Anticancer Agents	Phase 4	/	/
Hypoxia-inducible factor 1 alpha (HIF-2A)	PT2977^1^	/	Renal cell carcinoma	HIF inhibitors	Phase 3	/	/
Fibroblast growth factor receptor (FGFR)	Erdafitinib	/	Bladder cancer	Anticancer Agents	Approved	/	X

^1^ Data were extracted from clinicaltrials.gov (NCT04935359, NCT03496974, NCT04322188, NCT04347226, NCT00029042, NCT00880672, NCT04586231).

### 5.2 Peri-surgical interventions

As mentioned above, the possible mechanisms of surgery-related damage to ovarian reserve cannot be ignored. Accordingly, some measures have been taken to optimize the procedure to minimize the deleterious effect on ovarian reserve. A recent RCT randomized 200 women with unilateral OMA (≥5 cm) to receive bipolar coagulation or oxidized regenerated cellulose (ORC) during drainage or cystectomy for hemostasis (34). The trial showed that the use of ORC (drainage + ORC group and cystectomy + ORC group) significantly reduced recurrence rates, with minimal affection for the ovarian reserve in the drainage + ORC group. The use of ORC was generally safe, while encapsulation of fluid and foreign body granulomatous reaction had been reported (161). Some other RCTs also found that hemostatic sealant was non-inferior to bipolar coagulation for hemostasis during laparoscopic cystectomy for OMA patients and might be beneficial to preserve ovarian reserve (36, 162–164).

Similarly, to minimize the use of electrocoagulation and preserve ovarian reserve, some studies used vasopressin injection or epinephrine compress technique to reduce intraoperative bleeding, whereas there is a controversy as to whether this approach could preserve ovarian reserve. Alborzi et al. conducted an RCT to compare ovarian cystectomy after vasopressin injection in the mesovarium space (n=60) and direct cystectomy (n=60) for patients with unilateral OMA (3-6 cm) (165). The results showed that the control group had significantly higher hemostasis points and bleeding compared with the vasopressin group, but there was no difference between the two groups in postoperative serum levels of AMH and pregnancy outcomes. However, another retrospective cohort study indicated that for patients with bilateral OMA (>5cm), vasopressin injection could preserve ovarian reserve (166). An additional RCT revealed that the epinephrine compression method for ovarian stripping had the benefit of the preservation of the ovarian reserve, especially for those with OMA, which might be attributed to epinephrine ameliorated fibrotic changes and necrotic findings in the injured lesion (167). Importantly, no matter which surgical strategy is applied, the assessment of the ovarian reserve is crucial for counselling before the operation. And the patient should be fully aware of the effect of ovarian damage before proceeding to operation.

In addition, there are some studies discussing the impact of hormone therapy on ovarian reserve after cystectomy for OMA. A small single-center RCT compared two groups of women with OMA who received perioperative GnRHa treatment (n=22) or dienogest treatment (n=27) to study the effect on ovarian reserve (168). They found that dienogest was effective for preserving ovarian reserve by reducing the inflammatory response.

However, clinicians should aware that the different methods of peri-surgical interventions could be effective to reduce damage to the ovary, but the trauma still could not be fully reduced.

### 5.3 Assisted reproductive technology

Around 25-50% of infertile patients are diagnosed with endometriosis and up to 50% of women with endometriosis are referred to IVF centers for ART intervention (169). Although the mechanism of OMA-related infertility is unclear, previous studies suggested that the adhesion of the fallopian tube and ovarian (170), the oxidative damage on oocytes (171), and inflammation (172) might be responsible for it. In a systematic review and meta-analysis, Hamdam et al. investigated the impact of OMA on IVF/ICSI outcomes (173). The study showed that although the mean number of oocytes retrieved per cycle (MNOR) was lower and the cycle cancellation rate (CCR) was higher in women with OMA compared with those without OMA, the live birth rate (LBR) and the clinical pregnancy rate (CPR) were similar between the two groups. In subgroup analysis, women with OMA who received surgical treatment before IVF/ICSI had a similar CPR, LBR, and MNOR compared with those without surgical treatment. The results suggested that surgical treatment of OMA did not affect the IVF/ICSI treatment outcomes. Considering the surgical treatment of OMA might reduce ovarian reserve, physicians should weigh the pros and cons before stripping ovarian OMA prior to IVF/ICSI. HJ Park concluded that surgery prior to IVF was necessary when patients were suffering from severe dysmenorrhea or suspected of cancer. And when the size of OMA was very large, laparoscopic ovarian cystectomy could be considered before IVF (174).

Several studies compared GnRH agonist and GnRH antagonist ovarian stimulation protocols in women with endometriosis. An RCT found that the implantation rate and clinical pregnancy rate were similar in a GnRH antagonist cycle and a GnRH agonist protocol for women with stage I/II endometriosis and OMA (175). Drakopoulos et al. conducted a retrospective cohort study to compare long GnRH agonist with GnRH antagonist ART protocols for women with endometriosis (176). In patients with stage I-II endometriosis, the β-hCG positive, clinical pregnancy, and live birth rates were higher in the GnRH agonist group, but the difference was not statistically significant (P=0.07). No differences in pregnancy outcomes was observed between the two ovarian stimulation protocols in stage III/IV endometriosis group. Overall, there is no sufficient evidence to recommend the best ovarian stimulation protocol for OMA patients. More relevant clinical studies are required.

### 5.4 Fertility preservation

Fertility preservation (FP) has addressed massive attention since the development of reproductive technologies. FP is legislatively available in most European countries for patients with oncological, and benign diseases, as well as in transgender men (177). Cryopreservation of oocyte, embryo, and ovarian tissue can be applied together with potential medical and surgical interventions to preserve fertility. Oocyte and embryo cryopreservation requires ovarian stimulation while ovarian tissue cryopreservation (OTC) does not. Up to now, oocyte and embryo cryopreservation are preferable for women with age-related fertility loss, due to the advanced development of oocyte and embryo vitrification. Oocyte cryopreservation is usually for single women and embryo preservation is widely applied as a part of ART for married couples as the joint legal ownership with the male partner is necessary. OTC is an essential choice for patients who either have no sufficient time for ovarian stimulation or have adjacent tissue resected in a prior surgery. A combination of different approaches should be considered according to the individual’s situation (178).

As discussed above, both OMA itself and its surgical removal lead to reduced ovarian reserve with impaired yield and quality of oocytes. The preservation of fertility in patients diagnosed with OMA is especially important (179). However, there are limited data describing the effect of FP before surgical interventions in women with OMA so far (180). The first case of oocyte preservation in women with endometriosis was reported in 2009, which proposed the indication of FP in young women with severe endometriosis (181). Another publication reported the success of primordial follicle survival after ovarian tissue cryopreservation and transplantation in patients with severe endometriosis (182). However, these two studies had limitations to be presented as case reports. One observational study showed that FP for patients with a surgical history of OMA was related to poorer responsiveness of ovarian stimulation compared with OMA per se. The authors highlighted the importance of FP counseling before surgical resection in young women with severe endometriosis, however, there was no results of the FP results in healthy controls (183). The effect of OMA on controlled ovarian stimulation and the cumulative effect of stimulation on oocyte yield had been demonstrated in a research group in South Korea (184). Simultaneously, their study verified the efficacy of pre-operative FP in patients with OMA to prolong their fertility age (184).

There are several options for improving fecundities in patients with OMA, but FP should be with great potential for those with severe and repeated OMA. As OMA is still a novel topic in fertility preservation. It is conflicted about the timing, necessaries, approaches, and the patient’s willingness for the application of FP in those patients. The scenario is multifaceted, and both patients and physicians may be overwhelmed by the proper decision (185). Therefore, Marie-Madeleine Dolmans proposed an algorithm for fertility preservation in patients with endometriosis based on the strict indications, in which low level of AMH, age beyond 30 years, bilateral OMA, a high recurrence rate after surgery, OMA growing fast, and OMA at a young age should be taken into account (186).

## 6 Conclusion and perspectives

OMA is a prevalent disease in infertile women with a decreased ovarian reserve and impaired ovarian function. Surgery, the most common treatment of OMA, is disputable on the potential to destruct surrounding ovarian tissues. The interaction between OMA and ovarian aging can be found in numerous clinical cases, but there is no review to clarify the relevance and underlying mechanisms. Here we comprehensively summarised the clinical relevance and possible pathogenesis and mechanisms in commence of OMA and ovarian aging for the first time. Thereinto, fibrosis, inflammation, dysregulated angiogenesis, and oxidative stress may lead to the imbalance of DNA damage/repair and hyperactivation of primordial follicles, further resulting in a decreased ovarian reserve, which is not only an important characteristic of ovaries with OMA but also characterise the beginning of ovarian aging. The surgical removal of OMA also implies a detrimental effect on the surrounding ovaries, resecting the healthy ovarian cortex, hyperactivating primordial follicles, and thereby diminishing the ovarian function. Therapeutic targets on etiology pathways and molecules, i.e. PI3K/PTEN/Akt/FOXO3, TGF-β, IL-1β, IL-6, and TNF-α, are with the possibility to delay ovarian aging and restore fertility. Besides, modifications of surgical interventions like hemostasis methods are demonstrated to improve fertility loss to some extent but cannot absolutely avoid the detrimental effect of surgery. Fertility preservation is a recent-developed reproductive technology with great potential in maintaining fecundity in women with OMA. Due to the complexity and sophistication of this technique, more details, especially the different approaches to cryopreservation, the timing of tissue collection, ethical issues, and availability are needed to be discussed thoroughly before implying the application to patients.

Increased attention has been raised to seek an understanding of the pathophysiology and mechanisms of OMA leading to ovarian aging, which assists to propose new treatments and target therapy. However, many of these are still incompletely understood. We aimed to raise awareness of the missing pieces of puzzles and advocate more related studies. While the limited access to human samples, large-scale experiments on animals which share similar anatomy to human beings are also important. Novel animal models of OMA have been proposed recently, but they failed to manifest OMA exclusively. On account of the disparities of different subtypes of endometriosis, a proper animal model specific to OMA is the top priority, so to launch more related research for better management of OMA and its associated ovarian aging.

## Author contributions

ZT and CC participated in the research design. ZT participated in data evaluation, extraction, and interpretation. ZT, XG, and YL participated in the data validation and in the drafting of the manuscript. ZT, XG, YL, SH, JC, and CC critically revised the manuscript. All authors approved the final version of the manuscript.
